# Integrating Machine Learning with Flow-Imaging Microscopy for Automated Monitoring of Algal Blooms

**DOI:** 10.1021/acs.est.5c06078

**Published:** 2025-09-15

**Authors:** Farhan Khan, Benjamin Gincley, Andrea Busch, Dienye L. Tolofari, John W. Norton, Emily Varga, R. Michael McKay, Miguel Fuentes-Cabrera, Tad Slawecki, Ameet J. Pinto

**Affiliations:** † School of Civil and Environmental Engineering, 115724 Georgia Institute of Technology, Atlanta, Georgia 30332, United States; ‡ 647917 Great Lakes Water Authority, Detroit, Michigan 48226, United States; § Great Lakes Institute for Environmental Research, 8637 University of Windsor, Windsor, Ontario N9B 3P4, Canada; ∥ Khoury College of Computer Sciences, Northeastern University, Oakland, California 94613, United States; ⊥ LimnoTech, Incorporated, Ann Arbor, Michigan 48108, United States; # School of Earth and Atmospheric Sciences, Georgia Institute of Technology, Atlanta, Georgia 30332, United States; ∇ Brook Byers Institute for Sustainable Systems, Georgia Institute of Technology, Atlanta, Georgia 30332, United States

**Keywords:** flow-imaging microscopy, microalgae, HAB monitoring, background particle, out-of-distribution, freshwater ecosystems, openset classification

## Abstract

Real-time monitoring of phytoplankton in freshwater systems is critical for early detection of harmful algal blooms (HABs) to enable efficient response by water management agencies. This manuscript presents an image processing pipeline developed to adapt ARTiMiS, a low-cost automated flow-imaging device, for real-time algal monitoring in natural freshwater systems. This pipeline addresses several challenges associated with autonomous imaging of aquatic samples, such as flow-imaging artifacts (i.e., out-of-focus and background objects), as well as strategies to efficiently identify novel objects that are not represented in the training data set; the latter is a common challenge with the application of deep learning approaches for image classification in environmental systems. The pipeline leverages a random forest model to identify out-of-focus particles with an accuracy of 89% and a custom background particle detection algorithm to identify and remove particles that erroneously appear in consecutive images with >97 ± 2.8% accuracy. Furthermore, a convolutional neural network (CNN), trained to classify taxonomical classes, achieved 95% accuracy in a closed set classification. Nonetheless, the supervised closed-set classifiers struggled with the accurate classification of objects when challenged with novel particles, which are common in complex natural environments; this limits real-time monitoring applications by requiring extensive manual oversight. To mitigate this, three methods incorporating classification with rejection were tested to improve model precision by flagging irrelevant or unknown classes. Combined, these advances present a fully integrated, end-to-end solution for real-time HAB monitoring in natural freshwater systems, which enhances the scalability of automated detection in dynamic aquatic environments.

## Graphical abstract

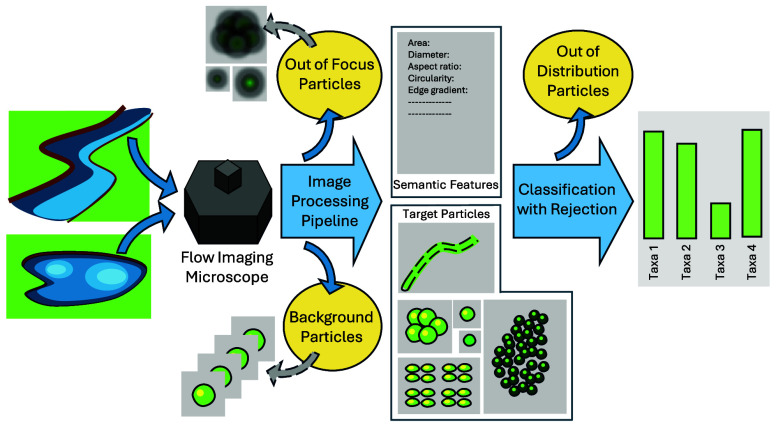

## Introduction

Cyanobacterial harmful algal blooms (cyanoHABs) are associated with toxin release and can also cause water discoloration, and taste and odor problems in the drinking water supply.^1–6^ Toxicity and hypoxia caused by these blooms can result in mortality to wildlife, loss of habitat for aquatic life, and economic losses for tourism and fisheries industries.^7–10^ To mitigate these effects, CyanoHAB monitoring is typically performed using manual microscopy, fluorometry, molecular assays, citizen science, or satellite remote sensing.^11–19^ In recent years, several emerging technologies have expanded the scope and resolution of HAB monitoring. These include uncrewed aerial vehicle (UAV) enabled aerosol collection for detecting phytoplankton in airborne particles,^20^ *in situ* hyperspectral reflectance,^21^ land-based video monitoring systems,^22^ and multimethod integration frameworks^23–25^ that combine field data with remote sensing. Taxonomic information along with biomass concentration is crucial for managing blooms and determining the appropriate response, as specific phytoplankton species are responsible for toxin production, taste and odor issues, and filter clogging at drinking water treatment plants (DWTPs). While manual microscopy offers detailed taxonomic information, it is time-consuming and requires expert taxonomists. Fluorescence probes and remote sensing can estimate phytoplankton biomass in bulk but lack the taxonomic detail necessary for actionable insights. Therefore, there is a pressing need for an automated imaging approach that is high-throughput and capable of providing quantitative taxonomic information.

There have been important advances in both imaging hardware and software such that distributed and automated real-time imaging for quantitative taxonomic data is likely feasible in the near future. Specifically, low-cost imaging solutions like LudusScope,^26^ SAMSON,^27^ HABscope,^28^ Openflexure Scope,^29^ PlanktoScope,^30^ ESPressoscope,^31^ GLUBscope,^32^ and ARTiMiS^33^ are available as alternatives to more expensive benchtop phytoplankton imaging platforms like FlowCam^34^ and CytoSense XR (CytoBuoy)^35^ or *in situ* instruments such as Scripps Plankton Camera,^36^ Imaging FlowCytobot (IFCB).^37^ Similarly, there have been significant advances in image processing as well. Existing commercial software, such as Visual Spreadsheet, uses statistical filters based on morphological properties to sort phytoplankton into different classification bins. However, this approach struggles to differentiate between phytoplankton with similar features (e.g., size).^38,39^ In contrast, advances in computing power, the availability of large data sets, and the popularization of convolutional neural networks (CNNs) have revolutionized image-processing approaches. This shift has popularized phytoplankton classification and significantly increased its accuracy.^40^ Research on phytoplankton classification surged over the past decade.^41^ Recently, Eerola et al. provided a comprehensive overview of research on automated plankton classification and identified some key associated challenges.^40^

Despite these advancements and the high classification accuracies reported in the literature, widespread automated plankton monitoring has not yet been realized. This is largely because a holistic solution, integrating both hardware and software, that can process environmental samples and provide taxonomy-resolved quantitative results on phytoplankton communities has yet to be developed for affordable, deployable devices typically accessible for water utilities for CyanoHAB monitoring. While such integrated solutions do exist for high-end systems like IFCB,^42^ these are often prohibitively expensive and not suitable for widespread deployment. Additionally, the classification models are typically tailored to specific locations or waterbodies and require extensive data collection and manual curation to develop representative training data sets, limiting their generalizability. In addition to classification challenges, automated phytoplankton monitoring using flow-imaging microscopy faces difficulties in processing images acquired under field-relevant conditions. which often include out-of-focus particles,^38^ background particles, abiotic particles, and out-of-distribution particles. Out-of-focus particles are particularly challenging to classify due to the lack of visual detail and distinguishing features. Background particles, typically those stuck in the imaging flow channel, may appear repeatedly across multiple frames, leading to duplicate counts.^43,44^ Often, manual processing is required to remove out-of-focus or background particles^44,45^ which is not consistent with the goal of automated imaging and image analyses. Out-of-distribution particles refer to those for which representative images are unavailable during the training phase, such as previously unseen plankton or non-plankton particles. This issue is especially prominent in natural aquatic systems where there is high diversity in plankton population.^46^ It is unrealistic to expect, at least currently, that the imaging platform will never encounter an out-of-distribution particle. Traditional CNN-based classifiers tend to misclassify these novel particles, making the classification process unreliable and again, necessitating manual curation. The requirement for such postprocessing has hindered the widespread adoption of flow-imaging microscopy for automated environmental monitoring.

This study focuses on the development of an image-processing pipeline that addresses some of the key challenges hindering automated plankton monitoring. Here, a low-cost (total bill of material cost $700 ± 100) miniaturized flow-imaging microscope (FIM), i.e., ARTiMiS,^33^ was used for sample processing and image collection. ARTiMiS was developed to make microalgal monitoring more affordable and accessible to a broader range of end users. Gincley et al.^33^ demonstrated that this device and its image-processing workflow can be used for real-time microalgal monitoring in cultivation systems designed for specific microalgae (i.e., low likelihood of encountering novel particles). In this study, ARTiMiS was adapted for phytoplankton monitoring, and an image processing pipeline was designed and developed to address challenges associated with environmental samples, reducing the need for manual intervention. Although this pipeline was specifically developed for ARTiMiS, the steps can be adopted for other automated image acquisition platforms.

## Materials and Methods

### ARTiMiS Flow-Imaging Microscope

ARTiMiS^33^ is a low-cost, self-contained, autonomous microscope that provides 5× magnification onto a Raspberry Pi Camera V2 (Sony IMX219 sensor) with a sample imaging resolution of 1.55 μm and a field of view of 725 μm x 545 μm, enabling detailed imaging suitable for environmental monitoring applications. At this level of magnification, a particle with a 5 μm diameter is represented by a 22.5-pixel image, providing sufficient detail for accurate classification and analysis of microscopic particles. Details of the magnification determination process have been described previously.^33^ The upper range of detection is limited by the size of the field of view. Illumination is provided by a programmable LED array enabling both bright and dark field illumination. An aqueous sample is circulated through a microfluidic chip with a 200 μm depth flow channel via a stop-flow mechanism. The device is equipped with autofocusing as an automatic routine before and during sample processing. A graphical user interface allows users to set parameters for a sample run, including settling time, illumination mode, number of images to be captured, autofocusing frequency, etc. A complete description of the instrument has been provided previously.^33^

### Collection of Plankton Images

Samples were collected by the Great Lakes Water Authority (GLWA) from the intake point of Lake Huron Water Treatment Facility, Water Works Park, and Southwest Water Treatment Plant (Figure S1) and shipped overnight to Georgia Institute of Technology weekly during summer months (May–September) and biweekly basis during winter months (October–April) from April 2022 to November 2023. Particle concentrations varied seasonally and were below the detection limit of the ARTiMiS (∼3.2 × 10^3^ particles/mL)^33^ during April and May. To mitigate this issue, samples were concentrated using a plankton net with a mesh size of 20 μm for 30 min at a flow rate of 6.8 L/min. Then the concentrate was eluted in 50 mL of water and prepared for shipping. Samples were also collected from Lake St. Clair at the Stoney Point Water Treatment Plant, Stoney Point, Ontario, Canada. These samples were collected from a tap installed at the intake well and concentrated from 218 L to 50 mL using a plankton net. A total of 154 samples were collected and run with the ARTiMiS instrument for data collection. In addition to the field samples, *Aphanizomenon* sp. (CAWBG01), *Planktothrix agardhii*, and *Dolichospermum flos-aquae*, all single-stranded, were cultured in the laboratory to generate a collection of high-priority cyanobacterial species impacting the Great Lakes region. These cultures were maintained in Jaworski medium at 25 °C under a 12 h:12 h day–night cycle at a photosynthetic active radiation (PAR) value of 80 μmol photons m^–2^ s^–1^. Cultures were grown in polycarbonate, unbaffled, sterile vent-cap flasks at nominal flask volumes of 125 mL (VWR International, 89095-258) and were agitated continuously using an orbital shaker. Field samples and laboratory cultures were imaged using ARTiMiS under both bright-field and dark-field modalities. A total of 30 to 90 images were collected for each sample, with a settling time ranging from 60 to 90 s before imaging. Autofocus was enabled for high-density samples and disabled for low-density samples.

### Development of the Image Processing Workflow

Figure S2 provides a complete overview of the developed image processing workflow and subsequent sections provide details for each step. The full workflow takes close to 9 s to process each widefield image with approximately 50 events using the onboard computer.

### Object Detection

Widefield images collected during the sample run were processed using an object detection algorithm (ODA) to detect particles and extract their cropped images. Here, a “particle” refers to any contiguous object detected within a specified region of interest (ROI), allowing for precise segmentation and examination of individual entities. Gincley et al.^33^ developed two ODAs for the ARTiMiS: of these, the Segmenter ODA was better suited for the variable particle sizes typically encountered in environmental samples and thus, it was selected for all image processing. Pixel coordinates and 47 geometric features were calculated for each detected particle. A description of the geometric features can be found in Table S1 of the Supporting Information.

### Out-of-Focus Particle Identification

An out-of-focus particle classifier was integrated into the image processing pipeline to identify and exclude out-of-focus particles from further analysis. This was a random forest (RF) classifier trained using the geometric features of the particles. For training and testing, a total of 3470 in-focus particles and 2663 out-of-focus particles were manually annotated and then divided into train and test sets at a 70:30 ratio. Pearson correlation analysis was conducted to detect and remove features with high autocorrelation, resulting in the elimination of 14 features. This resulted in the use of 33 features, which were used to train an RF model using the scikit-learn library in Python (v1.1.1). The features were then ranked in order of importance for class identity.^33^ Following this analysis, the model was retrained using the 12 top-ranked features to optimize its accuracy.

### Background Particle Detection

A two-stage background particle detection algorithm was developed to detect particles stuck in the field of view (FOV) in consecutive image frames. This algorithm clusters particles in three consecutive image frames based on their *x*–*y* pixel coordinates. Pairwise distances are calculated for every particle in the three consecutive frames. Particles with pairwise distances smaller than a user-defined threshold are clustered using the density-based spatial clustering of applications with noise (DBSCAN) method.^47^ A moving particle can sometimes be imaged within the defined threshold of a stationary particle. To address this, particles within a cluster based on the pairwise distance were grouped into subclusters based on their features. This resulted in subclusters consisting of particles that appeared at similar locations in the wide-field images and with similar, if not identical, features. Particles within subclusters were flagged as background particles, while particles outside of clusters were considered nonbackground or valid particles. In theory, this two-stage clustering method should be able to detect whether the same particle appears in two consecutive images. However, smaller debris may occasionally appear in the same ROIs, leading to measurement variation for the same particle. This can lead to incorrect identification of background particles: the particle with debris along with its duplicates in adjacent frames might be misclassified. To reduce misclassification, the algorithm flagged a particle as a background particle only when it was identified twice in three consecutive frames.

### Convolution Neural Networks

CNN architectures employed for the image classification task were based on the “micro-CNN” framework introduced previously.^33^ For this work, the CNN architecture was optimized through iterative evaluation of convolution layer configurations and dropout rates. The feature extraction pipeline was constructed using a sequence of convolutional layers, each followed by max-pooling and dropout operations. Configurations involving three, four, and five layers were explored to ascertain the optimal depth of the network. Convolution kernel sizes were also modified and evaluated. To ensure that a model was not overfitted to the training data set, varying dropout values were tested.^48^ After extensive hyperparameter tuning, the final model architecture was established as one block of CONV-MP, four sequential blocks of CONV-MP-DO, followed by two fully connected (dense) layers prior to model output. In this configuration, “CONV” describes a 2D convolutional layer, “MP” denotes a max pooling layer, and “DO” represents a dropout layer. The model employed a SoftMax activation function for output classification, and a dropout rate of 0.25 was used. During training, the Adam optimizer was used with a 0.001 learning rate and categorical cross-entropy as the loss function. The model configuration that provided the highest overall accuracy while reducing interclass accuracy disparity was chosen for this study. A similar architecture was used for out-of-distribution identification. The structure of the out-of-distribution classifier was a variation of the described model, also using SoftMax as the output activation function and trained with the Adam optimizer (0.001 learning rate, categorical cross-entropy).

### Out-of-Distribution Particle Identification

The SoftMax function in the final layer of a CNN generates probability scores for each class. The SoftMax thresholding method operates under the assumption that SoftMax scores for in-distribution (known) class images are high, whereas scores for ambiguous or out-of-distribution images are low. By establishing a cutoff score (threshold), it is possible to differentiate between known and out-of-distribution particles. In this study, particle images with SoftMax scores falling below the threshold were classified as out-of-distribution particles. Monte Carlo dropout (MCD), while sharing similarities with SoftMax thresholding in calculating probability scores, employs a distinct approach. Unlike SoftMax thresholding, MCD involves performing multiple forward passes through the network with varied dropout configurations to estimate model uncertainty. This variability in probability scores across different network configurations can assist in distinguishing out-of-distribution particles. For each particle image, 50 forward passes were executed, and the mean probability score was computed. The threshold for identifying out-of-distribution particles was then determined using the same methodology as with SoftMax thresholding. The third method, Class Anchor Clustering,^49^ leverages a distance-based loss function. This loss function trains the classification model to minimize intraclass distance while maximizing interclass distance. Theoretically, this should enhance the separation of out-of-distribution particles from the known classes by creating more distinct class boundaries.

### Train–Test Data set

Images were categorized into 12 distinct classes, with taxonomic labels including Chlorophyte unicell (*n* = 178), *Coelastrum* (*n* = 121), Cyanobacteria unicell (*n* = 627), *Micractinium* (*n* = 213), *Microcystis* (*n* = 201), *Merismopedia* (*n* = 189), *Pediastrum* (*n* = 243), *Scenedesmus* (*n* = 658), and *Staurastrum* (*n* = 58), where *n* indicates the number of images in each class. Due to the limited number of images available for individual diatom taxa, and considering their functional grouping as filter cloggers by water utility operators, diatoms from different taxa were aggregated into a single diatom class (*n* = 202). Colonies of small, round cells that are difficult to identify were aggregated under the “unidentified colony” class (*n* = 852). The filaments class (*n* = 385) consists of laboratory-grown cultures of *Aphanizomenon* sp., *Planktothrix agardhii*, *Dolichospermum flos-aquae*, and any filamentous phytoplankton from the field samples. They were aggregated as a single “filaments” class because it is challenging to differentiate among different filaments using our current flow-imaging configuration. A total of ∼4000 particle images across 12 classes were annotated and used for training, validation, and testing of image classification models. Examples of these classes are shown in Figure S3. These 12 classes were selected based on their high abundance in target sites and their functional importance, such as toxin production or filter clogging. Images from each class were divided into three subsets: train set, validation set, and test set in a 70:15:15 ratio. To enhance model generalization and increase the diversity of the training data, each image in the training set underwent augmentation. This augmentation was implemented as a set of 90° rotations and mirrors, resulting in 8 non-destructive transformations for each original image. This approach effectively expanded the training set and assisted in the learning of rotational invariance and feature robustness, improving model performance in classification tasks. The training and validation sets were used for model development, while the unseen test set was reserved for evaluating performance once training was complete. In addition to the 12 primary classes, “debris” and “novel” classes were annotated to test the model’s out-of-distribution particle identification capability. The novel class was formed with 250 images of phytoplankton not included in the training set, such as *Fragilaria*, *Dinobyron*, *Tetraspora*, *Stephanodiscus*, *Cymatopleura*, *Peridinium*, *Cosmarium*, and other rare taxa. The debris class (*n* = 643) consisted of various detrital particles with diverse shapes, annotated to represent non-planktonic material commonly encountered in environmental samples.

## Results and Discussion

### Ecosystem-Specific Calibration Improves the Performance of ODA for Freshwater Phytoplankton Imaging

Gincley et al.^33^ demonstrated that the “segmenter” object detection algorithm (ODA) outperformed the “fast object detector” in correctly processing images containing particles of varying sizes and morphologies. In this study, the efficacy of the segmenter algorithm was evaluated for accurately detecting phytoplankton in images from freshwater samples collected in the Great Lakes region. The segmenter algorithm was initially calibrated for algal communities in a full-scale microalgal cultivation system designed for nutrient recovery from wastewater. While the algorithm performed well for the majority of imaged particles, it encountered challenges with large colonial morphologies, resulting in the erroneous splitting of colonies into multiple objects. To address this limitation, the Segmenter algorithm was calibrated by adjusting its parameters to better accommodate the diverse cell types, sizes, and morphologies characteristic of freshwater samples. The object detection performance of the newly calibrated ODA was compared to the baseline performance (prior calibration) on samples with varying particle concentration (i.e., particles per unit volume) and FOV coverage; here, FOV coverage refers to the percent of the surface area in FOV that is occupied by particles. A dense (1.65 × 10^6^ particle/mL or 2.8% FOV coverage) and a sparse (4 × 10^5^ particle/mL or 0.7% FOV coverage) sample were chosen as such they closely resemble the density of samples [“EcoRecover medium density” (Eco Med) and “EcoRecover high density” (Eco High)] from the referenced data set used in the prior calibration. Multiple images for dense and sparse samples were selected, and objects in these images were annotated manually. For evaluation, each ROI identified by the ODA was classified into five categories: true positive (i.e., single particle in ROI), false positive (i.e., no particle in ROI), false negative (i.e., particle not entirely in ROI), merge (i.e., more than one particle in one ROI), and split (i.e., one particle distributed over more than one ROI).

The calibrated algorithm showed similar performance to the baseline algorithm for sparse samples (Figure 1A). The particle size distribution in sparse samples closely resembles that of the Eco Med sample (Figure 1B and C) with few large particles. This suggested that the performance of the calibrated model was as good as the baseline for particle distributions similar to EcoRecover samples.

**Figure 1:**
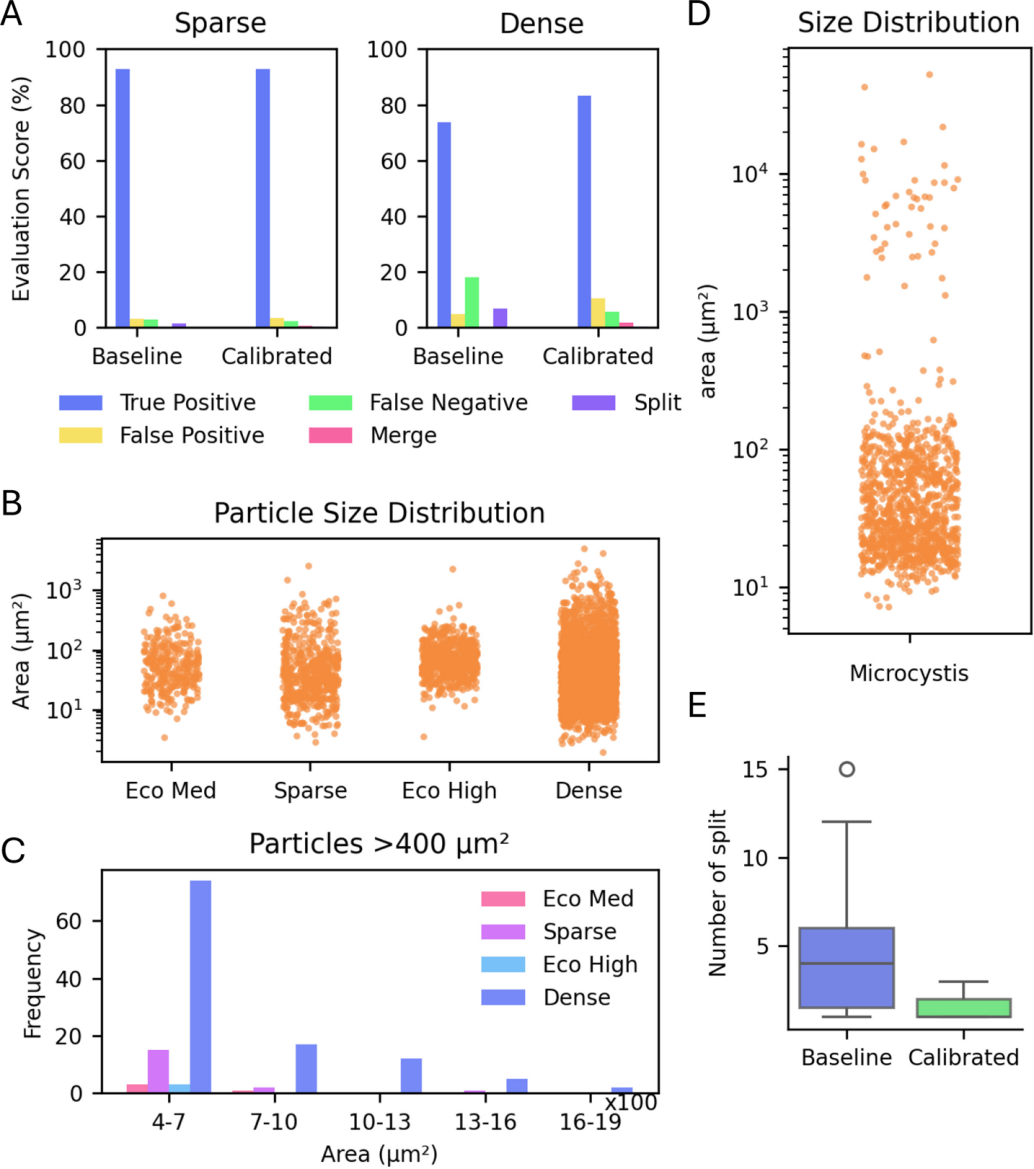
Particle size distribution and evaluation of particle identification for baseline vs calibrated ODA. (A) Performance comparison of baseline and calibrated ODA for sparse and dense samples. (B) Particle size distribution in EcoRecover medium and high-density samples and freshwater sparse and dense samples. (C) Histogram of particles with an area >400 μm^2^ in EcoRecover medium and high density and freshwater sparse and dense sample. (D) Particle size distribution for *Microcystis* colonies. (E) Number of splits performed by baseline and calibrated ODA for *Microcystis* colonies.

Dense freshwater samples contained relatively higher colonial or large particles (area >400 μm^2^) compared to the EcoRoecover data set (Figure 1B and C). Calibrated ODA achieved 83% accuracy for the dense sample whereas the baseline model exhibited 73.7% accuracy (Figure 1A). Calibrated ODA had more false positive incidents; however, among error types, false positives are of least concern given they can be identified as noise in downstream processing and discarded. On the other hand, calibrated ODA outperformed the reference baseline in terms of false negatives and split cases, an important reduction in error as these ROIs would otherwise be lost from analysis. Considering freshwater monitoring applications can encounter rare but impactful events, false negatives may lead to the failure to detect these occurrences. Surface water samples, including those in this study, often contain large plankton and colonies of plankton which are more susceptible to splitting during object detection. Thus, in the context of freshwater algae and cyanobacteria monitoring, minimizing the occurrences of false negatives and split cases is critical; the calibrated ODA performed similarly or better on these metrics (Figure S4A–D). The calibrated ODA was also tested on samples containing *Microcystis* colonies. *Microcystis* colony sizes can vary wildly, from 10 μm^2^ to more than 10^4^ μm^2^ (Figure 1D). When tested, the baseline ODA frequently splits these colonies into multiple smaller sections, or in some cases into individual cells, resulting in an overenumeration of single *Microcystis* colonies (Figure S4E and F). The calibrated ODA exhibited a much higher incidence of whole, intact colonial particle detection. Figure 1E highlights the split rate for each *Microcystis* colony by the two algorithms.

### Detection of Out-of-Focus and Background Particles Is Critical for Ensuring Accurate Quantitative Monitoring

Visual artifacts such as out-of-focus and background particles are inherent to flow-imaging microscopy.^43,50–53^ Following object detection, the pool of candidate ROIs to be identified contains both in- and out-of-focus particles. Examples of in-focus and out-of-focus can be found in Figure S5. To identify and exclude these out-of-focus particles from downstream processing, a preprocessing step was developed. A random forest (RF) classification model was trained on 33 semantic features of the in-focus and out-of-focus particles (collected during the 2022 sampling period), achieving 90% overall classification accuracy. Feature importance analysis (Figure 2A) revealed that parameters associated with edge noise and luminance intensity were most effective at describing distinct differences between the two classes. Feature selection was performed iteratively to maximize the inclusion of informative features and minimize the inclusion of features irrelevant to accurate class discrimination. The incorporation of 12 top-ranked features resulted in the highest overall accuracy (91%) with 92 and 90% correct predictions for in-focus and out-of-focus particles, respectively (Figure S7A). Figure 2B shows the density plot for the top four features, while density plots for all 12 features are provided in Figure S6. Some separation between in-focus and out-of-focus particle populations can be observed. As expected, misclassification predictions appear in the transition region of in-focus and out-of-focus particles where the feature distributions of the two classes overlap. A suspected cause for this is due to the inherent nature of degree-of-focus as being a continuous quality; the binary nature of the classification scheme necessarily imposes a cutoff threshold within the overlapping region, and complete accuracy is not achievable.

**Figure 2:**
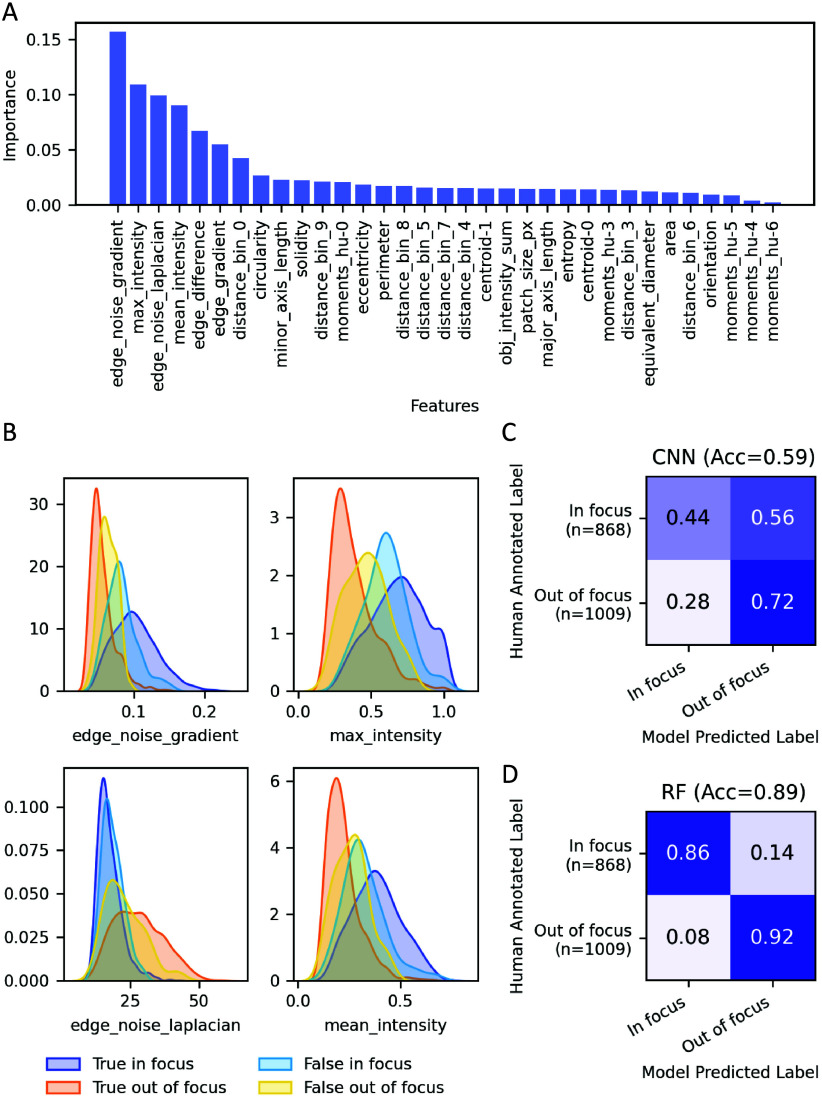
(A) Feature importance of the top 33 features relevant to in-focus and out-of-focus classification from the random forest (RF) classifier. (B) Density plot of the top four features, i.e., edge noise gradient, max intensity, edge noise Laplacian, and mean intensity, for true in-focus, true out-of-focus, false in-focus, and false out-of-focus particles. (C and D) Confusion matrices showing the classification performance of the CNN and RF models in identifying in-focus and out-of-focus particles.

In complement to the RF model, a CNN model was trained on the ROIs containing both in-focus and out-of-focus particles with the aim of investigating whether a more complex model, such as CNN, could potentially enhance accuracy by leveraging image-based feature extraction. The CNN model achieved almost identical results (92% accuracy) to the RF model (Figure S7B). However, when the model was tested on sample data collected the following year, it exhibited a high misclassification rate (only 59% accuracy, Figure 2C). In contrast, the RF classifier achieved 89% accuracy (Figure 2D) on the same data set, consistent with its performance on the data set from the prior year (Figure S7A). This suggested that the RF model is more generalized compared to the CNN model. Therefore, the RF model was chosen for out-of-focus identification for analysis of the complete sampling campaign.

In addition to out-of-focus identification, background particle detection is important for real-time continuous monitoring of HABs, especially for low-density samples. Due to the low concentration of particles of interest, even a small number of persistent background particles can significantly skew results. A naïve approach involves capturing blank images before and after a sample run to identify background particles; however, this method is unreliable since even blank images with ultrapure water can contain contaminating particles, leading to erroneous background particle counts.^45^ A number of dynamic scenarios may also occur: particles could get stuck in the FOV mid-sample run, previously stuck particles which may dislodge at a later time, or a stagnant particle may experience a changed degree of focus due to mid-run autofocusing; all of these scenarios can result in background decalibration. Thus, a static blank subtraction approach is not appropriate; instead, a dynamic approach is required to identify and remove background particles on a frame-by-frame basis. In theory, a background particle should appear in the same position in consecutive images. However, in a flow-through system, stagnant particles exhibit small shifts in position within the FOV during instrument operation as a result of fluid effects, small changes in camera or flow cell position, etc. These movements can also result in slight changes in the measured particle features. Successful removal of these stagnant background particles requires an approach that is tolerant to these perturbations in measurements.

A novel algorithm for background particle detection (BPD) was developed that tracks particles in successive frames based on their location and measured semantic features. The BPD algorithm was optimized for natural environmental samples and its performance was evaluated for low (1.6 × 10^5^ particles/mL), medium (3 × 10^5^ particles/mL), and medium-high (6 × 10^5^ particles/mL) density samples. First, 300 instances of stagnant particles and their corresponding coordinates were identified in images collected from the select sample runs. Travel distances were calculated for these particles from one frame to the next frame using their *x*–*y* coordinates. Mean and median travel distances were found to be 16.8 and 13.15 pixels, respectively, with a standard deviation of 11.5 pixels (Figure S8). The tolerance of the BPD algorithm was calibrated to detect stagnant particles with >95% accuracy even when the particles may have moved by up to 20 pixels (Figure 3A). Subsequently, the calibrated algorithm was used on images from sample runs (real images) as well as synthetic images. Synthetic images were created by seeding blank images with both background particles and target particles. To simulate low-, medium- and high-density samples, blank images were seeded with combinations of 10, 15, and 25 target particles and 3, 5, and 8 background particles, respectively. These incidence rates corresponded to 0.3, 0.5, and 0.8% FOV coverage and sample concentrations of 2 × 10^5^, 3 × 10^5^, and 5 × 10^5^ particles per mL, respectively. Target particles to seed synthetic images were randomly selected from the annotated ROI image library and placed at random *x*–*y* coordinates in each frame. For background particle seeding, identical copies of a random particle image were placed at randomly chosen positions on the images. Positions of the background particles were randomly jittered no greater than 12 pixels from frame to frame. On synthetic images, BPD achieved >99% accuracy with a small number of false positives and achieved 97 ± 2.8% accuracy for real images (from sample runs) with a small number of false negatives (Figure 3B and Figure S9).

**Figure 3:**
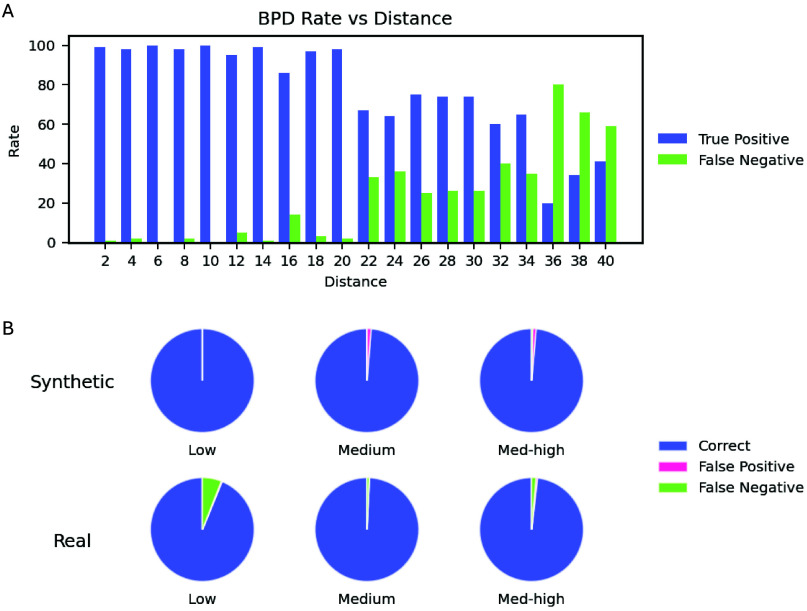
(A) Detection rate of background particles by the BPD algorithm at varying particle-to-particle distances. (B) Performance of the BPD algorithm on low-, medium-, and high-density real and synthetic samples.

### Closed-Set Classification Accurately Identifies Classes Included in the Training Data Set, but Performance Is Degraded for Out-of-Distribution Particle Types

Previously, Gincley et al.^33^ demonstrated that ARTiMiS could accurately classify particles from complex microalgal communities in a photobioreactor system and demonstrated that CNN models outperformed RF models for multiclass algal classification. Building on these findings, this study explored the application of CNN models for phytoplankton classification in freshwater samples. A custom CNN model was trained on the 12 primary classes, with the annotated data set split for training, validation, and testing. The optimized CNN model achieved an overall accuracy of 95%, with nine out of 12 classes exceeding 90% accuracy (Figure 4). The diatom and*Merismopedia* classes exhibited comparatively lower performance. In the case of Diatoms, misclassifications primarily occurred with the filaments class, likely due to the similar elongated morphology shared by both groups. For*Merismopedia*, confusion arose with the unidentified colony class, as both are composed of aggregates of small, round cells. Although the model misclassified only one *Staurastrum* image, the accuracy appeared disproportionately low due to the limited number of test images (*n* = 8). Overall, the micro-CNN model demonstrated strong performance in classifying the 12 most abundant phytoplankton classes observed in the field samples.

**Figure 4:**
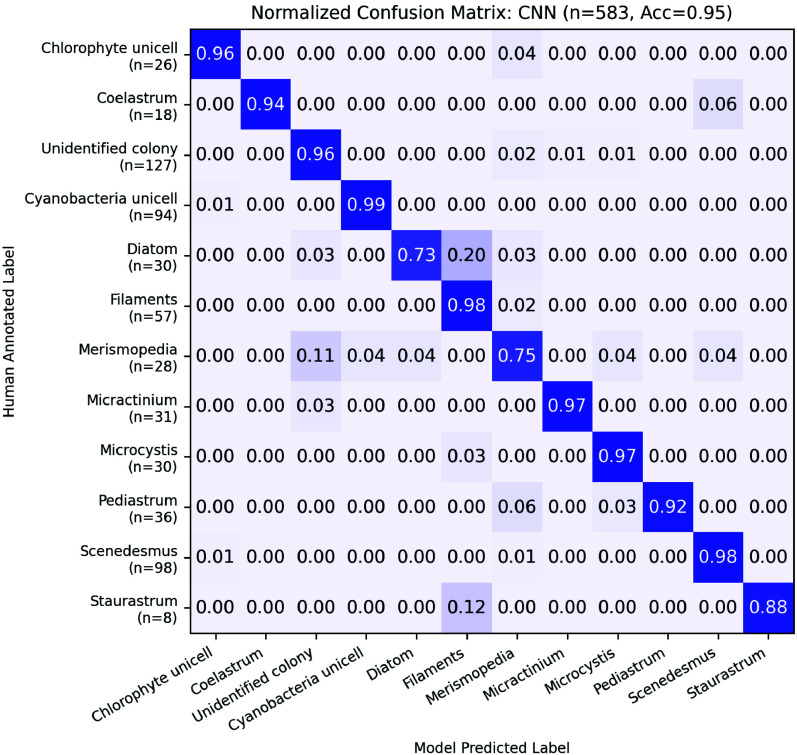
Confusion matrix showing performance of CNN model trained to distinguish among 12 classes of objects captured from environmental samples. Model accuracy, 95%, when evaluated on unseen annotated test data (*n* = 583 particle images).

A considerable proportion of phytoplankton classification studies benchmark machine- and deep-learning model performance on standardized public data sets, e.g., WHOI data set, ZOOSCAN data set, ISIIS PlanktonSet-1.0,^54–57^ or use meticulously curated internally maintained data sets.^58–60^ The most common class configuration is that of a “closed-set”, that is, a fixed number of predetermined classes selected due to their relevance to the respective study’s subject matter (e.g., Figure 4). It should be noted that such classifiers are only capable of assigning labels from this predetermined set, even in cases where a test sample is not represented in the original set. As a result, when deployed in scenarios where collected data include plankton classes in the original data set along with non-plankton objects and organisms not included in the original data set, classifier accuracy and precision underperform test-projected estimates in practice.^40,46,61^

To illustrate this point, a collection of particles (debris and novel class) was annotated but not included in the classifier training data set. Introducing these out-of-distribution objects into the test data set led to a decrease in accuracy from 95 to 59% (Figure 5A). Curating an exhaustive data set to represent natural systems (e.g., freshwater lakes) is currently impractical due to the labor (sampling and annotation) required. To begin to address the out-of-distribution-associated loss in model performance, a subset of the “debris” class representing particles outside of the standard set of phytoplankton taxa of interest, mostly non-planktonic particles, referred to as the “known out-of-distribution class” (KOC), was introduced to the data set prior to classifier training with the aim of reducing the classifier’s epistemic uncertainty. In theory, this would refine the decision boundaries associated with the original primary target classes, ultimately enhancing overall model performance. To investigate the effect of the KOC class on out-of-distribution object recognition, the remaining portion of the “debris” class and the novel class (Figure S10) was used as the test set. The KOC was strictly limited to the debris class, ensuring that the novel class remained entirely unknown to the model. With the inclusion of the KOC, classifier accuracy increased from 59% to 67% (Figure 5A) with 92% of the debris and 30% of the novel particles detected as out-of-distribution particles (Figure 5D and E). Figure 5B–E illustrates a two-dimensional view of the N-dimensional feature space encoded by the classification model using *t*-SNE,^62^ for classifier variants trained without (Figure 5B and C) and with (Figure 5D and E) the KOC. The KOC-trained variant exhibits greater inter-region distances and a higher proportion of out-of-distribution particles outside of class-associated regions, i.e., in the “unassigned” space. This behavior is advantageous in that fewer unknown out-of-distribution particles were misclassified as a primary target class, dropping from 100% to 8% for the debris class and to 70% for the novel class. While the inclusion of KOC improved the model’s robustness to unseen particles, the effect of intraclass morphological variability within the KOC class was not evaluated in this study but is an important aspect to be investigated further in future studies. In postprocessing, the objects classified as out-of-distribution particles can be assigned a null class label independent from the original class labels. By aggregating particles with this null label assignment, processed samples can then be reviewed by a human annotator to examine these specific particles.

**Figure 5:**
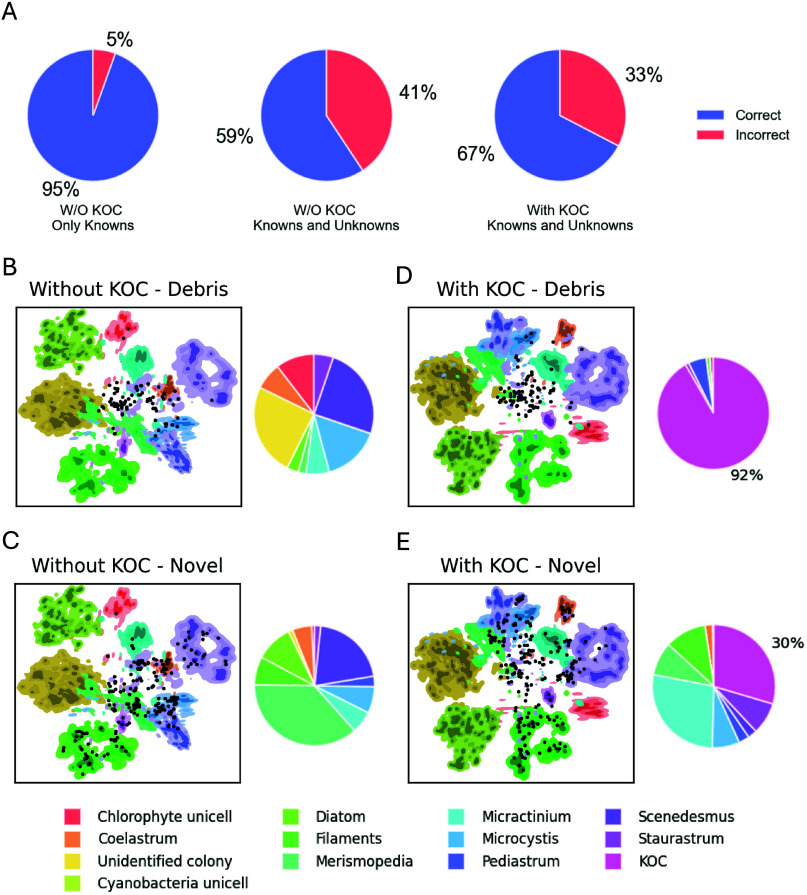
(A) Pie chart illustrating the prediction performance of CNN models trained with and without the KOC class on known and unknown particles. The percentages represent the fractions of correct and incorrect predictions for each model. (B–E) Density contour plot for models trained without (B and C) and with (D and E) the KOC, generated from *t*-SNE projections, with color-coded contour levels for each class in the training data set. Black points represent the “debris” class (B and D) and “novel” class (C and E) from the test set, showing its distribution across different class densities. The accompanying pie chart shows the fraction of the “debris” class and “novel” class classified as various classes for each model. The percentages represent the fractions of debris or novel class predicted as out of distribution particles (KOC).

### Classification with Rejection Using Probability Thresholding May Allow for Optimized Annotation of Novel Classes

Even with the inclusion of KOC, eliminating misclassifications is challenging due to the high diversity of planktonic microorganisms and non-cellular debris in samples analyzed from natural environmental systems. Given this complexity, phytoplankton monitoring in environmental samples aligns more closely with a “classification with rejection” or “open-set recognition” task where samples contain numerous types of both biotic and abiotic particles, often distinct from the classes the model was trained on. Previous studies^54,57,58^ have employed probability thresholding to mitigate misclassification. Probability thresholding involves only retaining predictions with a probability value higher than a given threshold. As the threshold increases, precision tends to increase as label assignment becomes stricter. Thus, the use of SoftMax thresholding can improve precision but at the cost of recall. Figure 6A shows the precision–recall curves for varying probability thresholds, comparing CNN models utilizing SoftMax thresholding both with and without the inclusion of the KOC during the training phase. Setting a threshold cutoff corresponding to 65% recall for the model trained with KOC (represented by the green curve) results in an overall precision exceeding 76%. Moreover, adjusting the threshold cutoff to achieve 60% recall can elevate precision beyond 80%. This approach may offer operational benefits by consolidating erroneous predictions into a single “out-of-distribution” class rather than distributing them across all classes, thereby simplifying manual verification. This increase in precision supports the validity of probability thresholding as an effective method for minimizing erroneous predictions in the autonomous monitoring of algae in freshwater environments.

**Figure 6:**
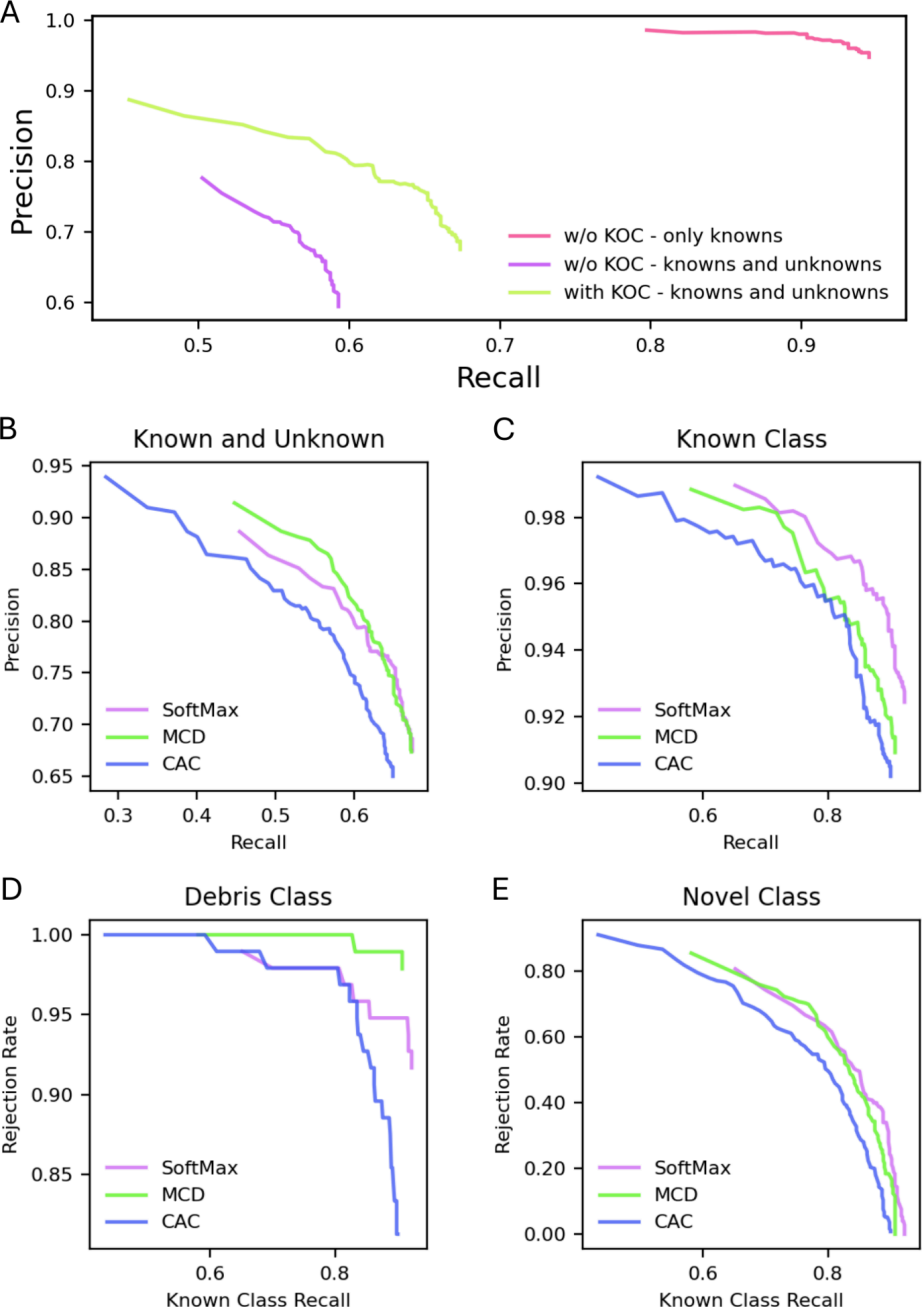
(A) Precision–recall curve for SoftMax models trained with (green) and without (red and violet) KOC on only known particles (red) and known and unknown (debris and novel) particles (green and violet). (B) Precision of SoftMax, MCD, and CAC models for varying recall values in both known and unknown class identification. (C) Precision–recall curve for the three models on the known class. (D and E) Rejection rate of the three models at varying known class recall values for the debris class and novel class, respectively.

However, as the threshold increases, a growing number of predictions are rejected or labeled as out-of-distribution, potentially leading to lower detection rates for low-density or rare particles. To further investigate precision and recall in the classification of non-curated data sets, two additional methods for detecting unknown out-of-distribution particles were explored: Monte Carlo dropout (MCD) and class-anchored clustering (CAC). The MCD method involves dropping out certain nodes during inference to generate multiple predictions for the same input, thereby estimating uncertainty by analyzing the variability in these predictions. In contrast, CAC is a distance-based open-set classifier that uses a distance metric to predict the class of a particle. This method is optimized to increase interclass distance while decreasing intraclass distance.^49^ To understand how rejection through thresholding affects the prediction precision, precision–recall analysis was done.

SoftMax and MCD demonstrate a similar relationship between precision and recall above 60% recall (Figure 6B). Below 60% recall, MCD shows a higher precision/recall ratio. On the other hand, CAC exhibits lower precision across all recall values. SoftMax has the highest precision vs recall ratio throughout the range and CAC has the lowest when focusing only on the known particles (Figure 6C). From this analysis, it is evident that the SoftMax model is better optimized for classifying known classes (closed-set classification). However, MCD showed better precision for below 60% recall. To understand this behavior, we looked at the rejection rate of the debris and novel classes for different known class recall rates (Figure 6D and E). MCD has the highest rejection rate for the debris class for any known class recall value. This may explain MCD’s higher overall precision below 60% recall. SoftMax has a higher rejection rate than CAC for known class recall above 80%, but below this value, SoftMax and CAC have similar rejection rates. For the novel class, SoftMax and MCD demonstrated similar rejection rates. In general, MCD demonstrated a higher out-of-distribution detection rate, while SoftMax performed best for known class classification. This suggests that no single method is definitively superior in all scenarios, and the choice depends on the specific use case. For applications requiring a high out-of-distribution identification or rejection rate, MCD would be the preferred method. Conversely, when a high yield of known class predictions is necessary, SoftMax would be more suitable. However, all three classification-with-rejection methods, CAC, MCD, and SoftMax thresholding, outperformed the closed-set classification approach, highlighting their overall effectiveness in handling out-of-distribution particles in environmental monitoring tasks.

### Toward Open World Recognition for Automated Classification of Phytoplankton in Natural Environmental Systems

Our findings suggest that integrating a known out-of-distribution class (KOC) into the classifier significantly enhances its ability to detect unknown particles and improves overall model performance. Classifiers typically struggle with out-of-distribution predictions due to epistemic uncertainty, often stemming from insufficient training data. By incorporating a known out-of-distribution class, this uncertainty is reduced, leading to more refined decision boundaries for known classes. Transitioning from a closed-set classifier to a classification model with a rejection option improves accuracy and precision while reducing misclassification rates. This approach also enables the classification model to identify and isolate particles with high prediction uncertainty, providing an opportunity for manual annotation and subsequent incorporation into the training data set, thereby increasing the model’s robustness. In practice, future efforts should concentrate on collecting more images of microalgae, phytoplankton, and other particles throughout the deployment lifespan of *in situ* monitoring devices like ARTiMiS and utilize classification-with-rejection methods to effectively differentiate between known and out-of-distribution particles. These out-of-distribution particles can then be categorized using unsupervised clustering methods or semisupervised classification methods followed by manual curation (Figure S11). Periodically updating the training data set with newly acquired images and retraining the classifier on this updated data set will reduce epistemic uncertainty and enhance the classification robustness for environmental monitoring; this approach, known as “open world recognition (OWR)”, was first defined by Bendale and Boult.^63^ An OWR system requires three tasks: classifying known and out-of-distribution particles, clustering out-of-distribution particles and labeling them as different novel classes, and applying incremental learning.^64,65^ This study suggests that OWR could be the optimal approach for real-time monitoring of freshwater phytoplankton, enabled by low-cost FIM platforms such as ARTiMiS, thus offering a dynamic and robust solution for managing the inherent variability of natural freshwater systems.

The pipeline developed in this study provides a generalizable framework for freshwater algae monitoring, as it addresses several critical challenges that are commonly encountered. Especially, the incorporation of background and out-of-focus particle detection enhances the quality of data acquisition and significantly reduces the burden of postprocessing, an important advantage in low cell density environments where irrelevant or noisy data can often obfuscate biological dynamics. Furthermore, the use of classification with rejection and the potential integration of open-world recognition not only improves classification accuracy but also enables the monitoring approach to adapt over time. This adaptability is essential for natural freshwater ecosystems, which often contain a highly diverse and dynamic particle population. These features make the pipeline well-suited for broader deployment in other freshwater systems, provided certain conditions, such as local calibration and preconcentration for very low-density samples, are met.

## Supplementary Material



## Notes

Sampling locations (Figure S1), image processing workflow (Figure S2), features measured by the feature extraction (Table S1), example images for each of the 12 primary classes (Figure S3), object detection by baseline and calibrated Object Detection Algorithm (Figure S4), examples of in-focus and out-of-focus particle images (Figure S5), density plot of top 12 features for true class, correct and incorrect predictions (Figure S6), confusion matrices for random forest and CNN-based out-of-focus detection (Figure S7), histogram of distances traveled by background particles in consecutive images (Figure S8), examples of background particle detection in real and synthetic images by the BPD algorithm (Figure S9), examples of out-of-distribution particles (Figure S10), and suggested open world recognition framework for automated monitoring of plankton in natural environments (Figure S11) (PDF)
