# Supplementary material for: Integrating Machine Learning with Flow-Imaging Microscopy for Automated Monitoring of Algal Blooms
Source: Environ Sci Technol. 2025 Sep 15;59(37):19885–98. doi: 10.1021/acs.est.5c06078 (PMC12461937; doi:10.1021/acs.est.5c06078)
Supplement: Supplementary file 1 [file es5c06078_si_001.pdf]

SUPPORTING INFORMATION

Integrating Machine Learning with Flow-Imaging Microscopy for Automated Monitoring of Algal Blooms

Authors: Farhan Khan[1], Benjamin Gincley[1], Andrea Busch[2], Dienye L Tolofari[2], John W Norton Jr[2], Emily Varga[3], R Michael Mckay[3], Miguel Fuentes-Cabrera[4], Tad Slawecki[5], Ameet J. Pinto[1,6,7] *

[1] School of Civil and Environmental Engineering, Georgia Institute of Technology, Atlanta, Georgia 30332, USA

[2] Great Lakes Water Authority, Detroit, Michigan 48226, USA

[3] Great Lakes Institute for Environmental Research, University of Windsor, Windsor, Ontario N9B 3P4, Canada

[4] Khoury College of Computer Sciences, Northeastern University, Oakland, California 94613, USA

[5] LimnoTech Inc, Ann Arbor, Michigan 48108, USA

[6] School of Earth and Atmospheric Sciences, Georgia Institute of Technology, Atlanta, Georgia 30332, USA

[7] Brook Byers Institute for Sustainable Systems, Georgia Institute of Technology, Atlanta, Georgia 30332, USA

* Corresponding author: Ameet J. Pinto (ameet.pinto@ce.gatech.edu)

Summary: 11 pages, 1 table, 11 figures

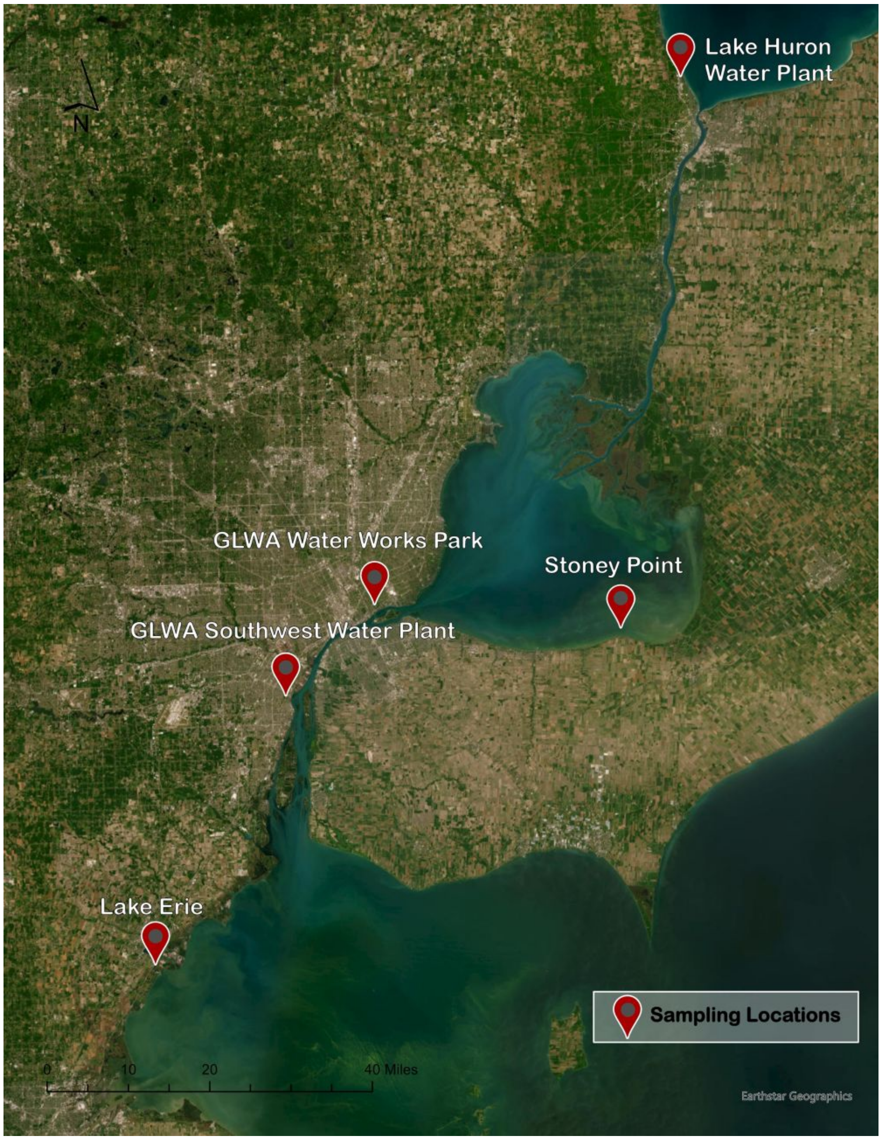

Figure S1: Sampling locations in Lake Erie and Lake St. Clair (Stoney point), raw water intakes in Detroit River for the GLWA Water Works Park and GLWA Southwest Water Treatment plant, and raw water intake in Lake Huron for the Lake Huron Plant.

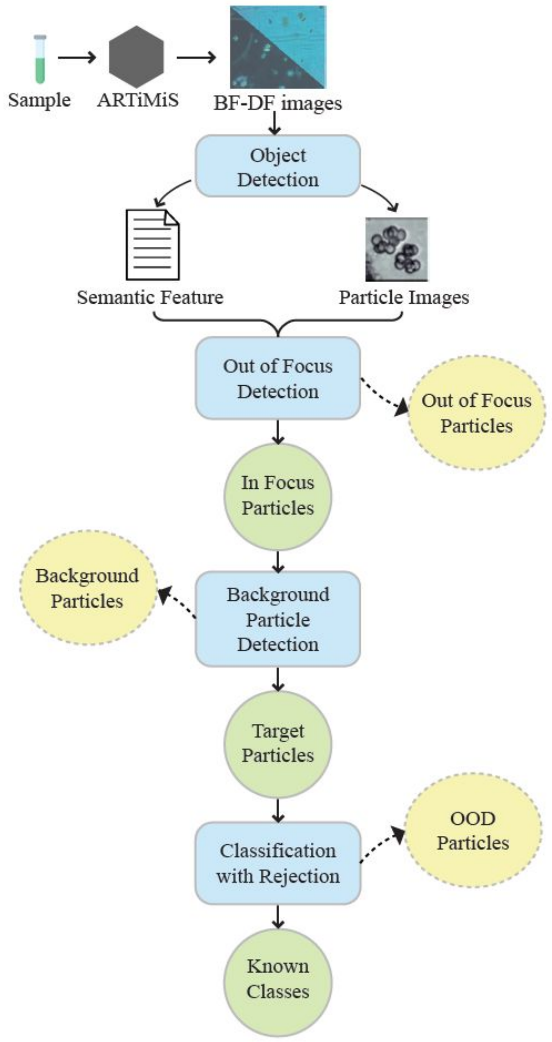

Figure S2: Schematic of image processing pipeline. Brightfield and darkfield images are collected using ARTiMiS. Object detection algorithm detects particles and calculates the semantic features of each particle. These particle images and semantic features are processed for out-of-focus particle designation, background particle detection, and classification with rejection steps sequentially to discard out-of-focus particles, background particles and out-of-distribution particles in the process. As a result of this processing, labels are assigned for each particle observed in the raw image dataset to be used in aggregate analysis.

Table S1: Features measured during semantic feature extraction. Features that are derived entirely or predominantly from an external computer vision library are indicated by dependency source. Features without a cited library dependency are derived using mathematical equations or methods from the Python standard library.

Feature Name	Description	Library Dependency	Feature used for RF	Feature used for BPD
UUID	Universally Unique Identifier, unique for each particle.	standard	No	No
XY Coordinates	Image coordinates of center of crop region.	standard	No	Yes
Area	Sum of count of pixels comprising dominant object in foreground.	scikit-image	No	Yes
Bounding Box Area	Area of the smallest rectangle enclosing the dominant object.	scikit-image	No	Yes
Convex Area	Area of the convex hull of the dominant object.	scikit-image	No	Yes
Total Area	Sum of all pixels in binarSy image foreground (can include other objects).	standard	No	Yes
Patch Size	Size of the image crop (ROI), in pixels and microns.	standard	No	Yes
Mean Intensity	Mean pixel brightness value across entire ROI.	scikit-image	Yes	Yes
Maximum Intensity	Maximum pixel brightness value across entire ROI.	standard	Yes	Yes
Object Intensity Sum	Sum of pixel brightness values for all pixels comprising the dominant object.	standard	No	Yes
Total Intensity Sum	Sum of all pixel brightness values across the entire ROI.	standard	No	Yes
Solidity	Ratio of pixels in the enclosing region to pixels of the convex hull image.	scikit-image	Yes	Yes
Perimeter	Perimeter contour calculated through the centers of border pixels using a 4-connectivity.	scikit-image	No	Yes
Equivalent Diameter	Diameter of a circle with the same area as the region.	scikit-image	No	Yes
Feret Diameter (max)	Maximum Feret's diameter, the longest distance between points around the convex hull.	scikit-image	No	Yes
Major Axis Length	Length of major axis of an ellipse having the same normalized second central moments as the dominant object.	scikit-image	No	Yes
Minor Axis Length	Length of minor axis of an ellipse having the same normalized second central moments as the dominant object.	scikit-image	Yes	Yes
Eccentricity	Ratio of the focal distance over the major axis length, calculated from an ellipse having the same normalized second central moments as the dominant object.	scikit-image	No	Yes
Orientation	Angle between the 0th axis and major axis of the ellipse having the same normalized second central moments as the dominant object.	scikit-image	No	Yes

Centroid	Weighted centroid of the image.	scikit-image	No	Yes
Hu Moments	Image moments: translation, scale, and rotation invariant.	scikit-image	Yes	Yes
Hu Circularity	Circularity of object using the 0th Hu's moment as the object's radius.	scikit-image	No	Yes
Entropy	Shannon's entropy of the image.	scikit-image	No	Yes
Circularity	Circularity of object using the object's calculated area and perimeter.	standard	No	Yes
Euler Number	Euler characteristic of binary image.	scikit-image	No	Yes
Object Topography	Distance transform of object, binned as topographic levels.	scipy	No	Yes
Aspect Ratio	Ratio of minor axis length to major axis length.	standard	No	Yes
Biovolume (Sphere)	Calculated volume of object, assuming sphere (circular) geometry.	standard	No	Yes
Biovolume (Spheroid)	Calculated volume of object, assuming spheroid (elliptical) geometry.	standard	Mo	Yes
Edge Noise (Laplacian)	Blurriness of object as calculated as the standard deviation of Laplacian of Gaussian blur.	OpenCV	Yes	Yes
Edge Noise (Gradient)	Blurriness of object as calculated as the standard deviation of Sobel gradient of Gaussian blur.	OpenCV	Yes	Yes
Edge Gradient	Visual gradient at edge of object, calculated as average pixel intensity of object's outer edge.	standard	Yes	Yes
Edge Difference	Visual gradient at edge of object, calculated as difference in pixel values outside and inside the object's edge.	standard	Yes	Yes

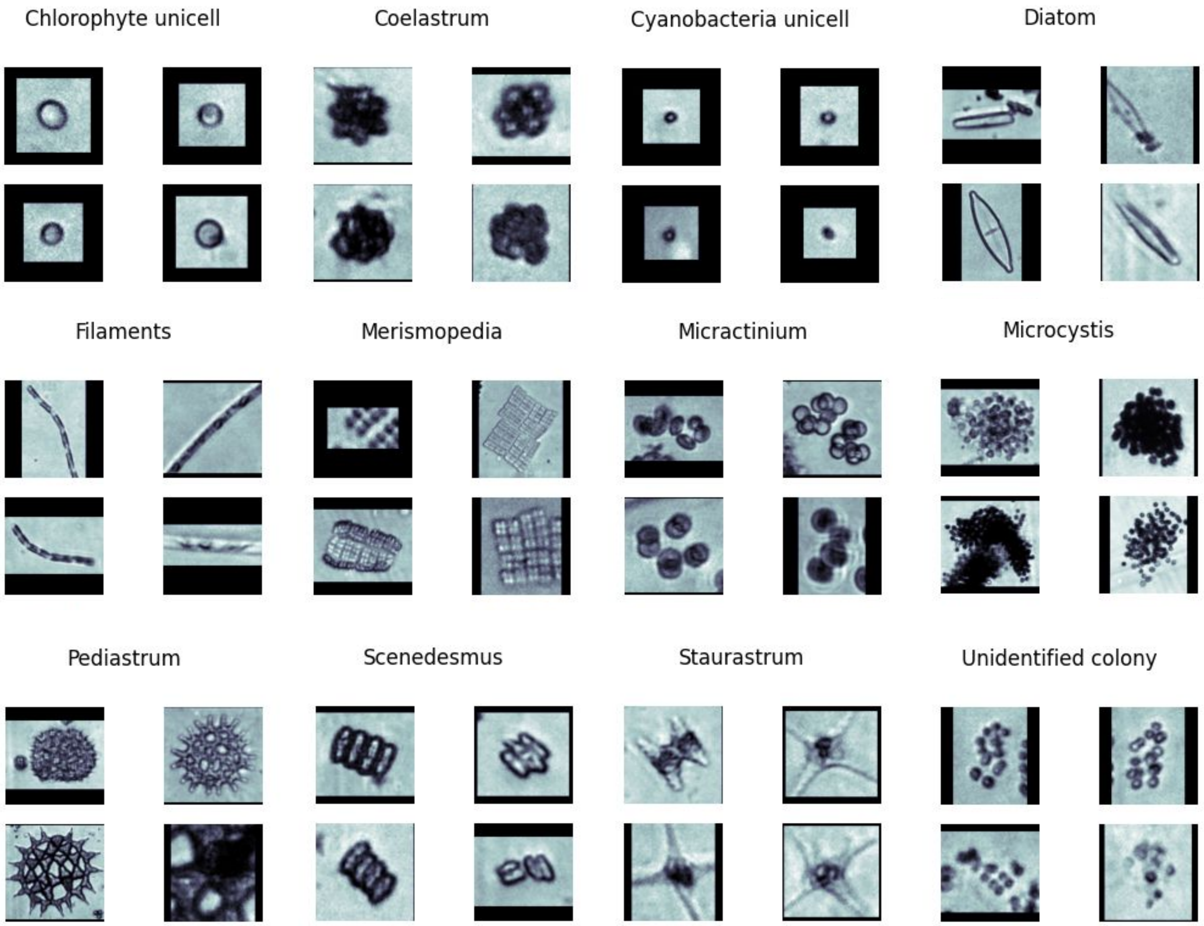

Figure S3: Four example images for each of the 12 known classes

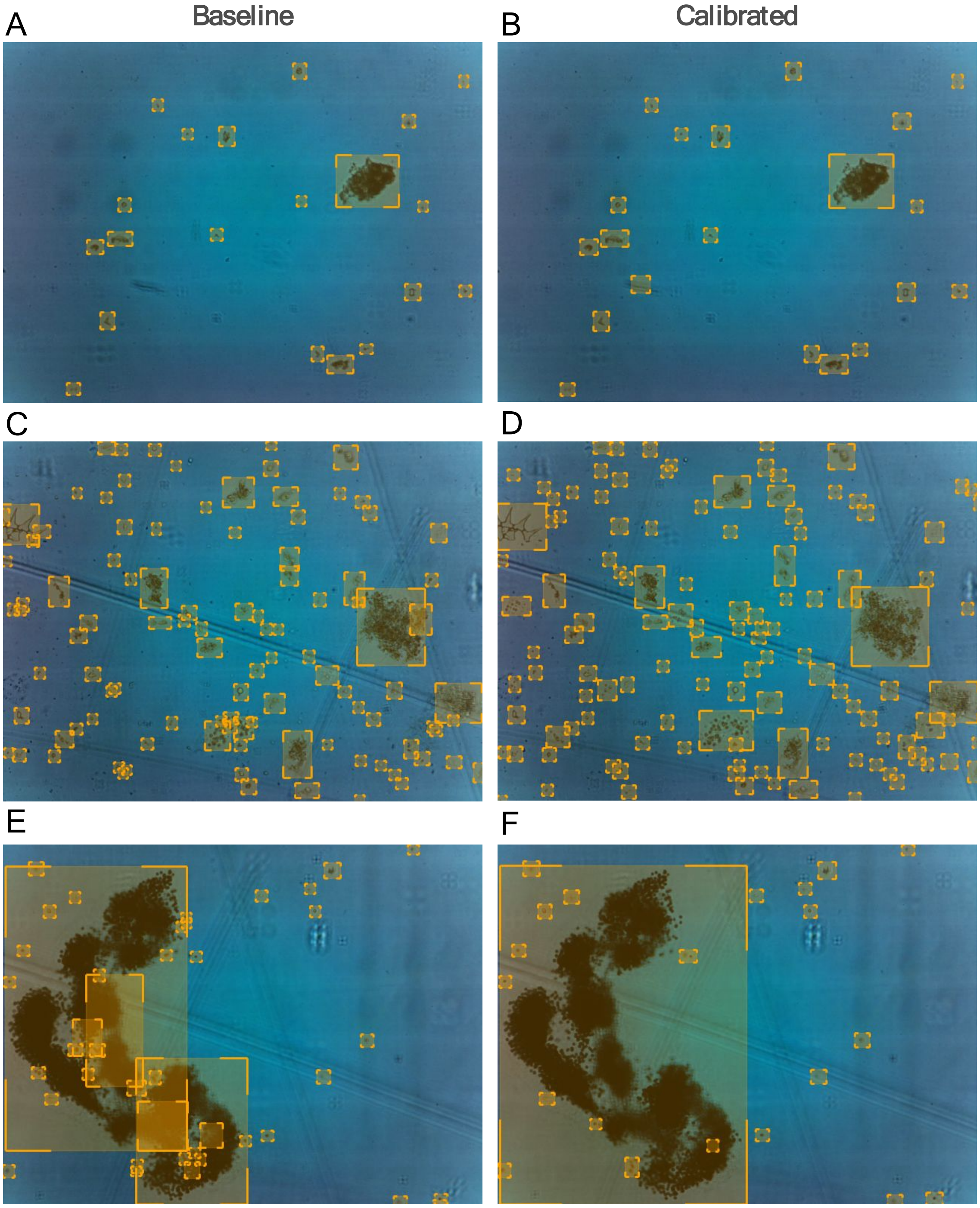

Figure S4: Objects detected in widefield images by baseline (A, C, E) and calibrated (B, D, F) ODA. Yellow boxes represent individual objects (either single cells or colonies). (A, B) show image samples for freshwater sparse samples, (C, D) represent dense samples, and (E, F) display image samples for Microcystis colonies.

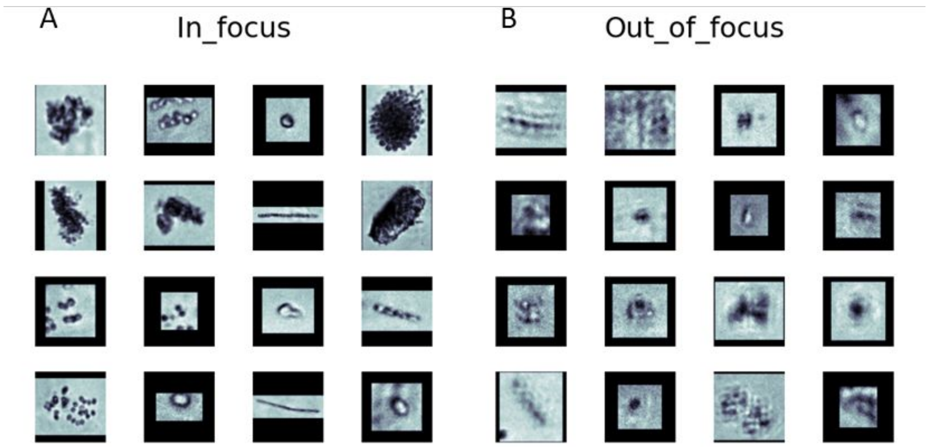

Figure S5: Examples of A) in-focus and B) out-of-focus particles

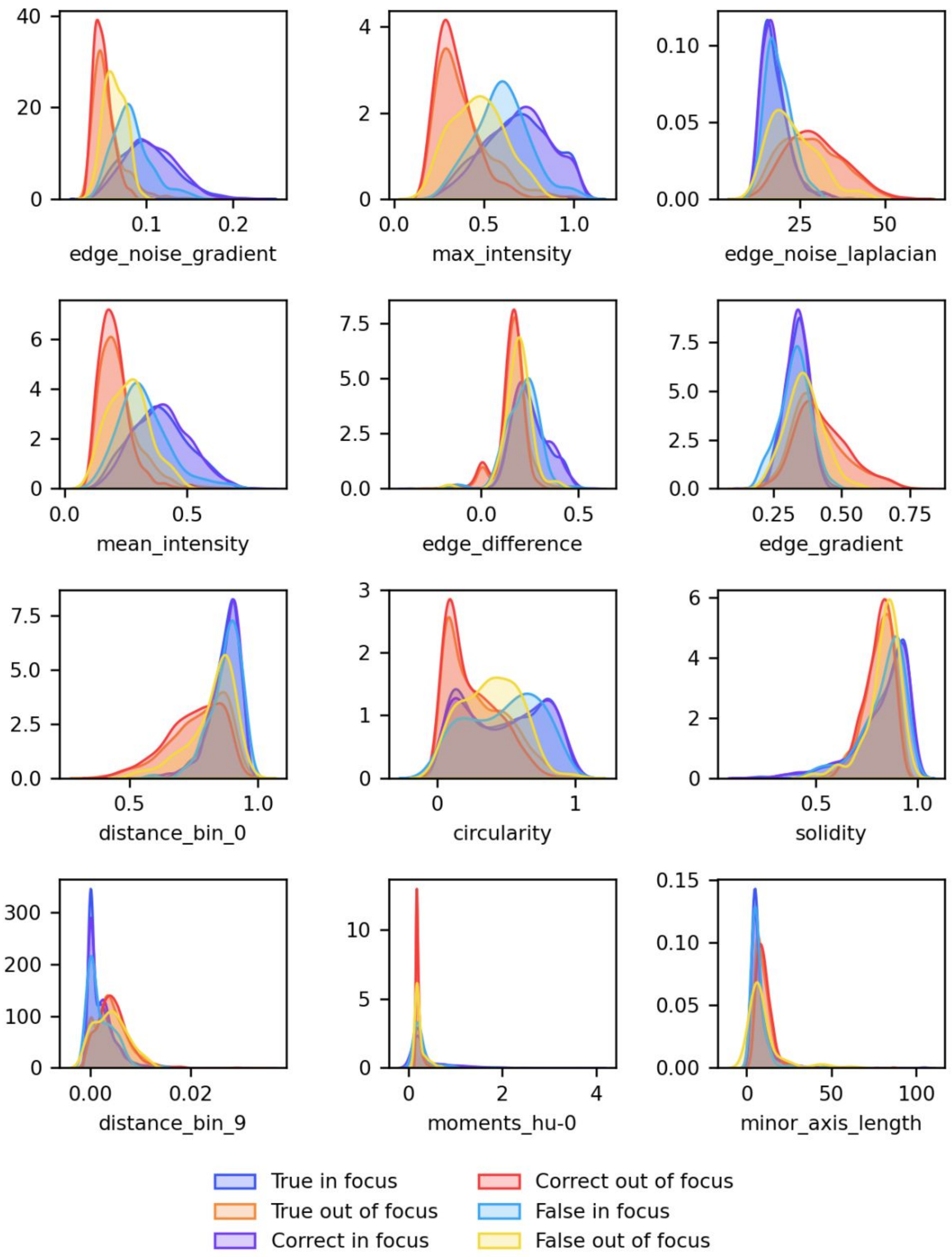

Figure S6: Density plot of top 12 features for true class, correct and incorrect predictions

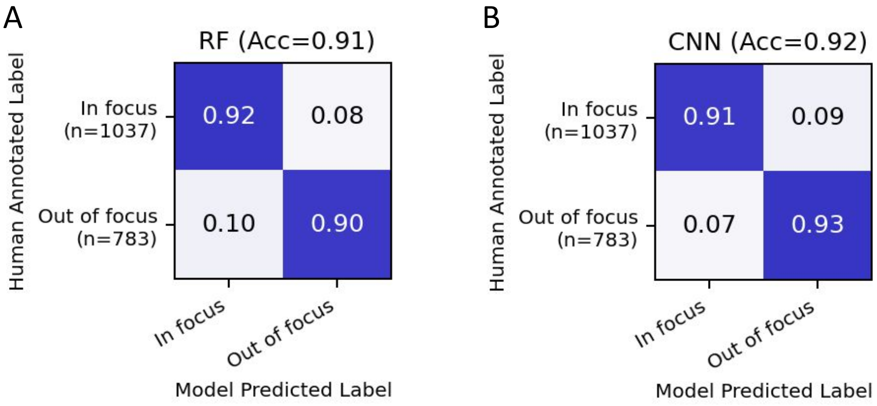

Figure S7: Confusion matrices showing performance of A) random forest, B) CNN based out-of-focus detection

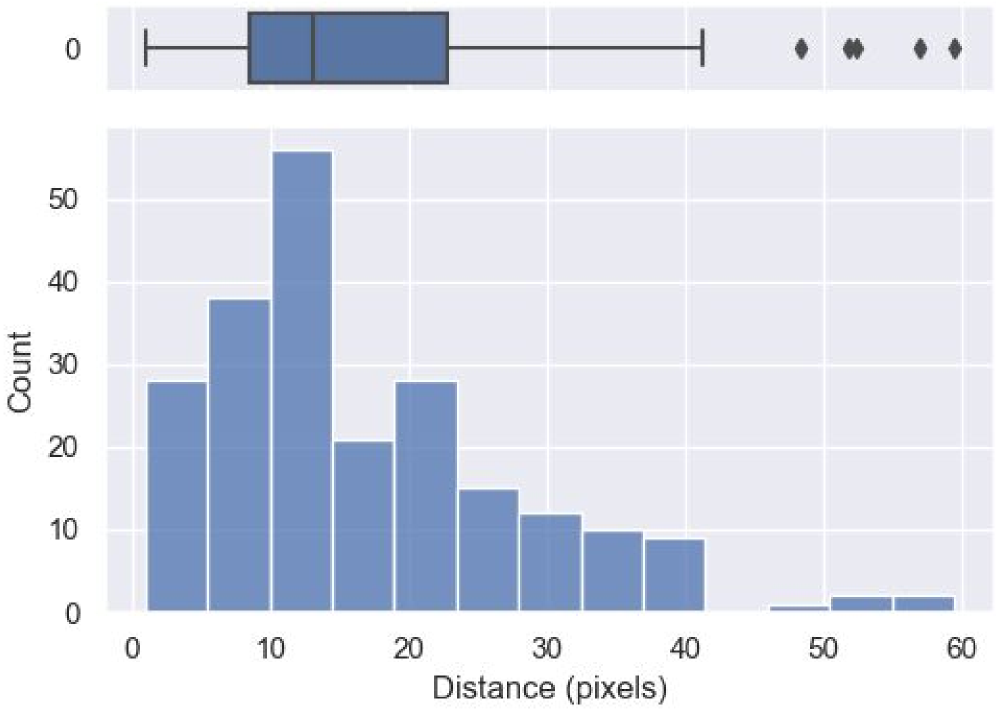

Figure S8: Histogram of distances traveled by background particles in consecutive images

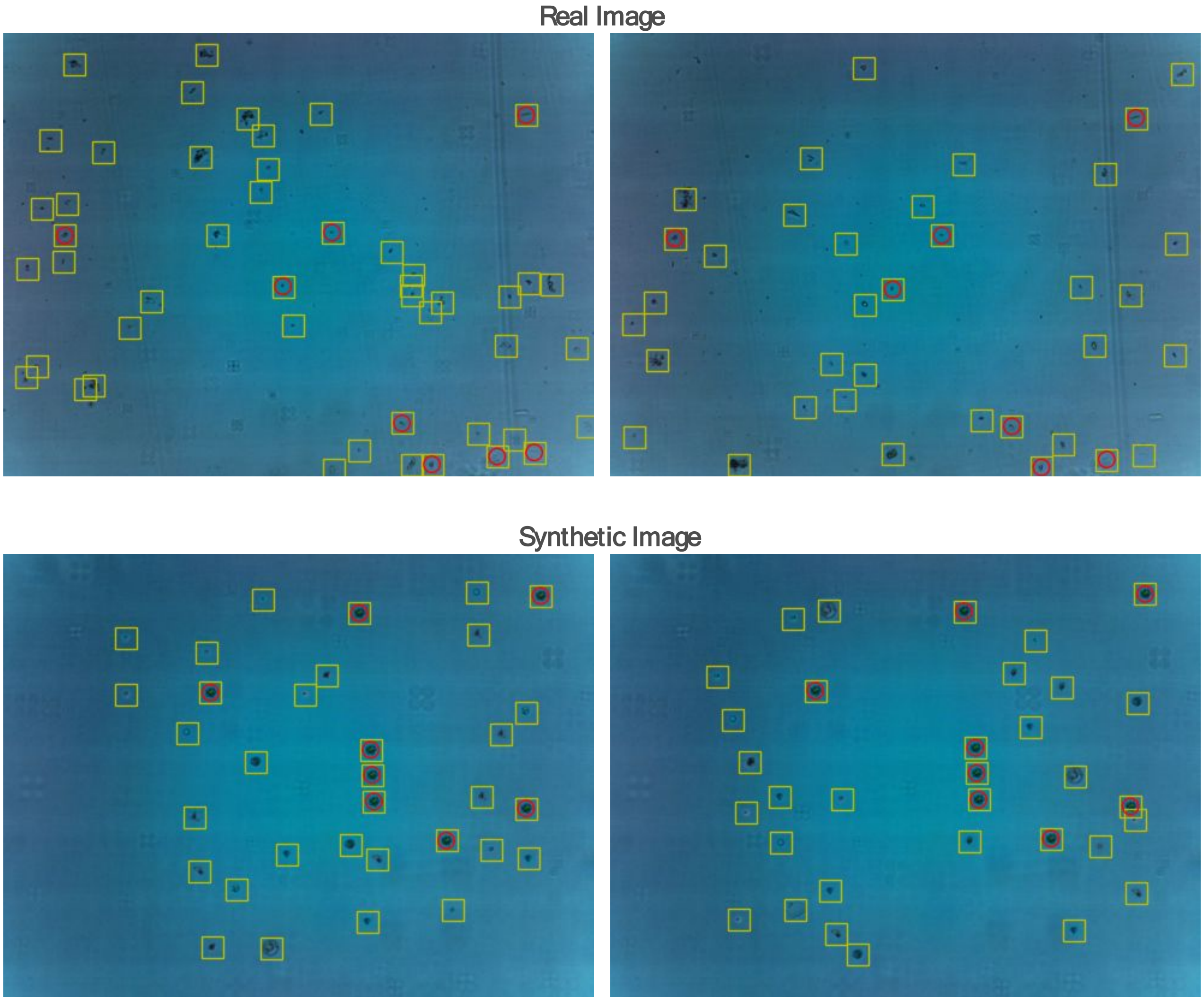

Figure S9: Background particle detection by BPD algorithm on real images and synthetic images (yellow box represents particle detected by object detector and red box represents particles identified as background particle in two consecutive images)

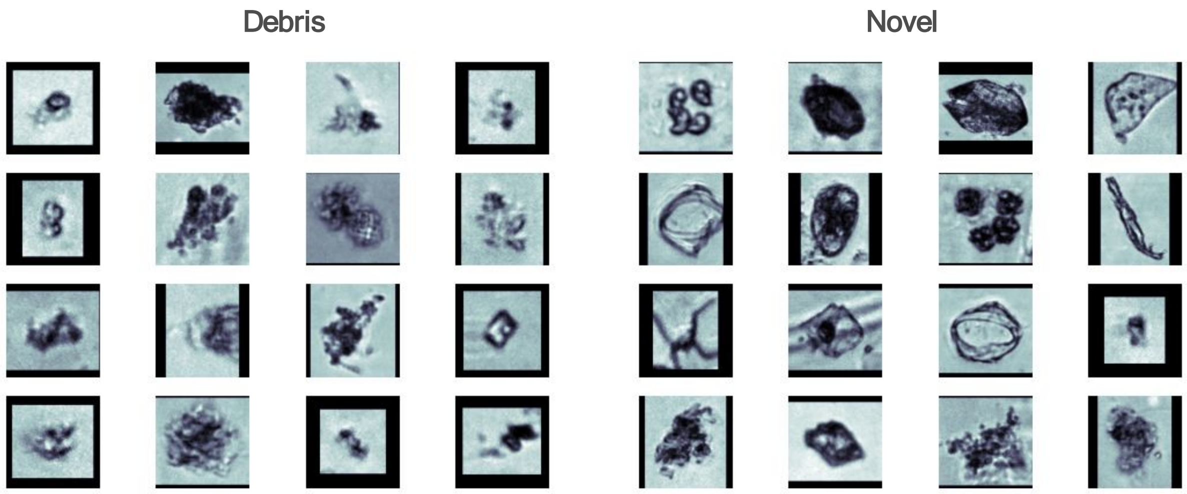

Figure S10: Example images out-of-distribution particles: debris on the left and novel on the right

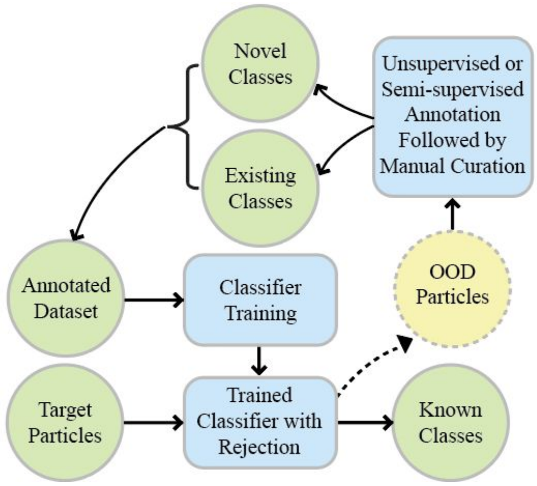

Figure S11: Suggested OWR framework for automated environmental monitoring system. Out-of-distribution (OOD) particle images, identified through classification with rejection, require re-annotation and integration into the annotated dataset for continuous improvement of the system
